# Vocational Aspirations, Cognizance and the Availability of Career Counselling Among Dental Interns in Bhubaneswar City, India: A Cross-Sectional Survey

**DOI:** 10.7759/cureus.89720

**Published:** 2025-08-10

**Authors:** Spandita Das, Arpita Singh, Gunjan Kumar, Kabir S Dash, Ipseeta Menon, Kunal Jha

**Affiliations:** 1 Department of Public Health Dentistry, Kalinga Institute of Dental Sciences (KIDS) Kalinga Institute of Industrial Technology (KIIT) Deemed to be University, Bhubaneswar, IND

**Keywords:** apprehensions, dental students, dentistry, motivation, vocational guidance

## Abstract

Background

Career paths, choices, and overall professional contentment of dental students are likely shaped by the vocational advice, skill sets, and practical experiences they acquire during their dental school tenure.

Aim

To determine the career aspirations, apprehensions and to assess the availability of career guidance among dental interns of three private dental colleges in Bhubaneswar city, India.

Methods

A cross-sectional survey was conducted among 207 dental interns of three private colleges of Bhubaneswar city using a self-structured English questionnaire. The questionnaire consisted of 24 questions divided into four sections, which included demographics, vocational aspirations and cognizance for choice of dentistry as a career and availability of career guidance. Data were collected, analyzed and was represented as numbers and percentages using the Chi‑square test. The level of significance was set at p<0.05.

Results

A total of 198 dental interns responded to the questionnaire. There was a slightly higher representation of women (59.60%) compared to men (40.40%). A larger proportion of women (96, 62.34%), compared to men (58, 37.66%), reported that dentistry was not their initial career choice. There was a notable difference in the preference for becoming a specialist dentist between men and women (p=0.008), with 56 (60.22%) women aspiring for specialization compared to 37 (39.78%) men.

Conclusion

A majority of the participants expressed that a low pay scale represents the primary limitation in the field of dentistry as a profession. Moreover, most participants aspire to continue in the field of dentistry, with the majority opting for postgraduate studies after completing their internship.

## Introduction

An essential consideration towards a bright and optimistic prospect for the future pertains to selecting a profession to pursue [1]. The relevance and necessity of choosing the appropriate professional path cannot be overstated. Multiple considerations, for instance, those pertaining to earnings and perks, professional stability and prestige, personal or manual skill utilization and a keen interest and curiosity in the fields of science and research, could potentially have an impact on this decision-making process [2]. It can be challenging to define and assess one's professional satisfaction at times and in certain situations due to its complicated nature and dependence on numerous variables [3].

Considering dentistry as a career path has the potential to offer numerous perks such as independent work, dignity, prestigious status socially and professionally, higher standards of living, higher-than-average earnings and novel opportunities [1]. Aspirations, perspectives and assumptions of students and graduates fluctuate with time; therefore, it's critical to examine how students and interns at a dental college envision their future problems in light of the significant time and financial commitment they will be making [4]. Future dentists will work in a field where globalization and revolution are happening at an accelerated pace [5].

In India, there are 329 dental colleges, including a notably large proportion of private institutions, with about 35,025 openings for dental students to pursue the course; there are 3,26,736 registered dentists [6]. The number of dentists in India is increasing by about 8% annually, whereas the country's population is growing by 1.31% annually. Despite the fact that the Indian state of Odisha has a dentist-to-population ratio of 1:1,000,000 as opposed to the WHO-recommended ratio of 1:7500, about 3000 dentists are waiting for regular appointments in the state [7].

People have diverse priorities and perspectives about their professions due to their varied socioeconomic status. Sometimes individuals choose careers based on the recommendations of their parents, older siblings or both. Consequently, they might find themselves working in a field where they lack expertise, adding to the challenges they face in their work [4]. Considering that dentistry serves as a healing discipline, studies on the rationale behind dental career choices have revealed that altruism is the mainstay of motivation [8].

The present-day dentistry graduates nevertheless encounter hurdles to balance the rising number of undergraduate seats with the declining number of merit seats and the rising cost of postgraduate expenses. On the other hand, dental graduates are under a great deal of strain due to a lack of dental employment possibilities in India, the growth of new private dental clinics and the lack of restructuring of professional credentials abroad. Most students are unaware of the plethora of possibilities that have lately become available after the completion of BDS (Bachelor of Dental Surgery). Furthermore, to increase career prospects and opportunities and broaden the scope of the Indian oral health profession, policymakers, administrators and educators must adopt a renaissance approach [9].

The factors influencing individuals' decisions to pursue dentistry have been extensively examined across various nations [10]. Students are presented with a multitude of career options, making it challenging for them to identify the best path that aligns with their passions and income expectations. It's important to recognize that dentistry, like any other professional career, evolves in response to advancements in skills, continuous innovation in techniques and the introduction of new materials. This underscores the importance of being adaptable and responsive to challenges, as it enables individuals to achieve a progressive career trajectory [11].

Dentistry holds a distinctive position in society as a professional healthcare field, yet it remains an emerging discipline in India [12]. Despite the varied reasons for opting for dentistry, the common expectation of a secure and promising professional future serves as a significant motivator for most students throughout their dental education journey [13].

It is very important for the undergraduates to understand their motives for choosing dentistry, the challenges and their views concerning the future of their profession so as to preserve a motivated workforce. In a state like Odisha, which has achieved academic and social advancements, it is of utmost importance to comprehend the perspectives of dental students in order to foster the expansion of the healthcare industry. There is limited research conducted on this subject in India, resulting in uncertainty regarding the motivations behind students choosing to become dentists. Only limited information about dental students from the state of Odisha is currently available. The purpose of this survey was to find out what influences dental interns in Odisha when it comes to choosing dentistry as a profession of choice, and career expectations and to assess their career prospects in the future upon completion of the BDS programme. Therefore, this study sought to assess the vocational aspirations, cognizance and availability of career counselling among dental interns at three private dental colleges in Bhubaneswar City, Odisha, India.

## Materials and methods

A cross-sectional, questionnaire survey was conducted to assess the career aspirations, apprehensions, and availability of career guidance among dental interns at three private dental colleges in Bhubaneswar city. The survey was carried out from March to April 2024 after receiving approval from the Institute Ethics Committee (IEC) of Kalinga Institute of Medical Sciences, Bhubaneswar.

According to the data obtained from all the three colleges, there were 66 interns in Kalinga Institute of Dental Sciences (KIDS), Bhubaneswar; 67 in the Institute of Dental Sciences (IDS), Bhubaneswar; and 75 in Hi-tech Dental College. Since universal sampling was adopted, all dental interns from the three dental colleges in Bhubaneswar city were included in the study. However, a minimum sample size was estimated using the G*Power software, version 3.1.9.7 for Windows (free to use software developed by Heinrich-Heine-University and downloaded from https://g-power.apponic.com/). The input parameters included a two-tailed test, an effect size of 0.32, a significance level (α) of 0.05, and a desired power (1-β) of 0.95. Based on these values, the required total sample size was calculated to be 116 participants. The analysis also yielded a non-centrality parameter (δ)=3.6378, a critical t-value=1.9810, and degrees of freedom (df)=114. The actual power achieved was 0.9502.

The inclusion criteria included interns from the private dental colleges who were present during the data collection period and who consented to participate in the study. Subjects who were not dental interns or were unwilling to participate were excluded. A self-structured questionnaire, consisting of both open-ended and close-ended questions, was used to quantify the responses of the participants. The questionnaire, along with an information and consent sheet, was manually distributed to the participants. Those who agreed to participate signed the consent form and completed the questionnaire. Socioeconomic status was assessed using the modified Kuppuswamy scale [14].

The questionnaire consisted of 24 questions (six open-ended and 18 close-ended) divided into four sections: demographics, vocational aspirations for choosing dentistry as a career, cognizance of choosing dentistry as a career, and availability of career guidance. The internal consistency of the items was measured and found to be satisfactory (Cronbach alpha=0.857). Additionally, the questionnaire was pilot-tested on 20 dental interns to reduce ambiguity and misinterpretation of the questions.

Data were entered into the Statistical Package for Social Sciences (SPSS), version 27.0 (IBM Corp., Armonk, NY). Descriptive analysis was performed to calculate the frequencies of categorical variables. The inferential statistics comprised the Chi-square test and Mann-Whitney test, with the level of significance set at p<0.05.

## Results

Out of 207 dental interns, 198 completed the questionnaire with a response rate of 95.2%. Table 1 demonstrates the sociodemographic characterization of the study population. A slight female predominance was observed, and the participants were fairly evenly distributed across the three participating dental institutions, namely KIDS, IDS, and Hi-Tech Dental College and Hospital, ensuring balanced institutional representation. The admission year of all interns was 2019. The enrollments in the three different colleges were evenly distributed, with approximately one-third of interns from each institution. The majority of interns reported an urban background, with comparatively fewer from periurban areas and very limited representation from rural settings. Only a small proportion reported having one or more dentists in the family, indicating limited direct professional exposure prior to entering the field.

**Table 1:** Sociodemographic Characteristics of the Study Population

Characteristics	Frequency (N)	Percentage (%)
Gender		
Male	80	40.40
Female	118	59.60
College		
Kalinga Institute of Dental Sciences	64	32.32
Institute of Dental Sciences	63	31.82
HI-Tech Dental College and Hospital	71	35.86
Place of upbringing		
Urban	150	75.76
Periurban	46	23.23
Rural	2	1.01
Socioeconomic class		
Upper	78	39.4
Upper middle	118	59.6
Lower middle	2	1
Number of dentists in the family		
Nil	159	80.30
1	22	11.11
>1	17	8.59

The present study found that dentistry was not the initial career choice for a majority of the participants (Table 2), with 62.34% of women and 37.66% of men indicating that they had considered other options. Although a higher proportion of women reported dentistry as a secondary choice, the difference between genders was not statistically significant (p=0.141). Similarly, when asked about their motivation for choosing dentistry, over half of both men (50.79%) and women (49.21%) cited personal interest, with no significant gender difference (p=0.064). In terms of decision-making, 60.90% of women and 39.10% of men acknowledged exploring alternative careers before finalizing dentistry, though this trend was not statistically significant (p=0.472). Regarding the initial impressions upon entering the BDS program, both male and female respondents reported a range of experiences. Common responses included feelings of satisfaction, dissatisfaction, ignorance and other mixed emotions. While certain emotional trends appeared more frequently in one gender over the other, these patterns did not reflect statistically significant differences.

**Table 2:** Distribution of Vocational Aspirations BDS: Bachelor of Dental Surgery.

Questions		Men, n (%)	Women, n (%)	Total, n (%)	p-value
Vocational aspirations					
Was dentistry your first choice as a career?	Yes	22 (50%)	22 (50%)	44 (100)	0.141
	No	58 (37.66)	96 (62.34)	154 (100)	
What motivated you to study dentistry?	Own Interest	32 (50.79)	31 (49.21)	63 (100)	0.064
	Parents' pleasure	19 (48.72)	20 (51.28)	39 (100)	
	Positive image of dentist/dentistry	11 (39.29)	17 (60.71)	28 (100)	
	Relatives’ recommendation	5 (41.67)	7 (58.33)	12 (100)	
	Friends’ recommendation	4 (28.57)	10 (71.43)	14 (100)	
	Financial advantages	0 (0)	4 (100)	4 (100)	
	Desire to help/serve people in my community	9 (23.68)	29 (76.32)	38 (100)	
Did you have any alternatives to dentistry before opting dentistry as a career?	Yes	61 (39.10)	95 (60.90)	156 (100)	0.472
	No	19 (45.24)	23 (54.76)	42 (100)	
What was your first impression when you joined BDS?	Satisfied	37 (38.95)	58 (61.05)	95 (100)	0.692
	Unsatisfied	21 (40.38)	31 (59.62)	52 (100)	
	Ignorant	14 (50.00)	14 (50.00)	28 (100)	
	Others	8 (34.78)	15 (65.22)	23 (100)	
What is your opinion regarding dentistry as a profession?	Helps to make money	29 (34.12)	56 (65.88)	85 (100)	0.226
	Increase my social standing	27 (42.19)	37 (57.81)	64 (100)	
	Serve people	24 (48.98)	25 (51.02)	49 (100)	
Over the past few years of education and practice, has the course given you more interest towards dentistry?	Yes	67 (42.95)	89 (57.05)	156 (100)	0.160
	No	13 (30.95)	29 (69.05)	42 (100)	

Table 3 shows that 109 participants agreed and 28 participants strongly agreed that there is development and advancement in the field of dentistry. However, there is no significant difference between men and women in agreeing or strongly agreeing with the development and advancement in dentistry (p=0.590). Regarding job stability, a higher percentage of women (53, 59.55%) agreed or strongly agreed that a job or career from BDS would provide steady employment compared to men (36, 40.45%), though the difference is not significant (p=0.137).

**Table 3:** Distribution of Cognizance BDS: Bachelor of Dental Surgery.

Questions		Men, n (%)	Women, n (%)	Total, n (%)	p-value
Cognizance					
Do you agree there is development and advancement in the field of dentistry?	Strongly disagree	11 (50.00)	11 (50.00)	22 (100)	0.590
	Disagree	7 (53.85)	6 (46.15)	13 (100)	
	No opinion	11 (42.31)	15 (57.69)	26 (100)	
	Agree	42 (38.53)	67 (61.47)	109 (100)	
	Strongly agree	9 (32.14)	19 (67.86)	28 (100)	
Do you agree that a job or career from BDS will give you steady employment?	Strongly disagree	4 (28.57)	10 (71.43)	14 (100)	0.137
	Disagree	15 (57.69)	11 (42.31)	26 (100)	
	No opinion	20 (42.55)	27 (57.45)	47 (100)	
	Agree	36 (40.45)	53 (59.55)	89 (100)	
	Strongly agree	5 (22.73)	17 (77.27)	22 (100)	
What do you think is the major limitation in dentistry as a profession?	Increasing professional competition	21 (43.75)	27 (56.25)	48 (100)	0.476
	People neglecting oral health or taking it lightly	25 (43.86)	32 (56.14)	57 (100)	
	Job insecurity	20 (42.55)	27 (57.45)	47 (100)	
	Low pay scale	14 (30.43)	32 (69.57)	46 (100)	
In your opinion, what is the biggest challenge you might face after completion of undergraduation?	Financial Constraints	11 (33.33)	22 (66.67)	33 (100)	0.377
	Pursuing post-graduation	17 (37.78)	28 (62.22)	45 (100)	
	Restricted Job Opportunities	37 (41.11)	53 (58.89)	90 (100)	
	Attracting and retaining patients	1 (20.00)	4 (80.00)	5 (100)	
	Increasing cost of dental materials and equipment	14 (56.00)	11 (44.00)	25 (100)	

Table 4 shows that a large proportion of respondents expressed interest in continuing their journey in dentistry beyond the undergraduate course, indicating a strong inclination towards further professional development. Notably, significant gender differences were observed in two key areas. First, women were more likely than men to rely on faculty members for career guidance, with this difference being statistically significant (p=0.023). Second, the aspiration to pursue specialization in dentistry was more prominent among female respondents, also showing a significant gender-based variation (p=0.008).

**Table 4:** Distribution of Availability of Career Guidance BDS: Bachelor of Dental Surgery.

Questions		Men, n (%)	Women, n (%)	Total, n (%)	p-value
Availability of career guidance					
Do you want to continue in the field of dentistry after undergraduation?	Yes	60 (37.50)	100 (62.50)	160 (100)	0.087
	No	20 (52.63)	18 (47.37)	38 (100)	
What is your preference after BDS?	Post-graduation	37 (34.26)	71 (65.74)	108 (100)	0.065
	Private Practice	11 (44.00)	14 (56.00)	25 (100)	
	Migrating Abroad	8 (33.33)	16 (66.67)	24 (100)	
	Government jobs (Defence/ Railways)	8 (50.00)	8 (50.00)	16 (100)	
	Salaried jobs (Teaching jobs/Hospitals)	6 (66.67)	3 (33.33)	9 (100)	
	Non-clinical career options (MHA/MPH/Pharmacovigilance)	6 (50.00)	6 (50.00)	12 (100)	
	Statistician	4 (100)	0 (0)	4 (100)	
What are the available career guidance options?	Faculty	20 (31.25)	44 (68.75)	64 (100)	0.023
	Program/Workshops	18 (60.00)	12 (40.00)	30 (100)	
	Seniors and Friends	24 (53.33)	21 (46.67)	45 (100)	
	Family	9 (36.00)	16 (64.00)	25 (100)	
	Mass Media/Social Media/Internet	7 (26.92)	19 (73.08)	26 (100)	
	Education consultancies	2 (25.00)	6 (75.00)	8 (100)	
What kind of dentist would you like to be in the future?	General dentist	22 (52.38)	20 (47.62)	42 (100)	0.008
	Specialist	37 (39.78)	56 (60.22)	93 (100)	
	Researcher	1 (14.29)	6 (85.71)	7 (100)	
	Academician	2 (9.09)	20 (90.91)	22 (100)	
	Others	6 (50.00)	6 (50.00)	12 (100)	
	Undecided	12 (54.55)	10 (45.45)	22 (100)	

Table 5 presents a z-score of -1.081 with a p-value of 0.2799, showing no statistically significant difference between the responses of the two groups. This implies that gender did not significantly influence agreement levels concerning the competencies provided by the BDS program for job security. Similarly, a z-score of -1.569 and a p-value of 0.1166 indicated no significant gender difference regarding opinions on career development and advancement in dentistry. Additionally, a z-score of -1.467 and a p-value of 0.1425 suggest that gender did not significantly affect perceptions of stable employment with a BDS degree.

**Table 5:** Mann-Whitney U Test for Comparing Agreement

Gender	N	Rank Sum	Expected Rank Sum	z score	p value
Vocational Aspirations: Do you agree BDS has given the required competencies for professionals to avail a job at the present day?					
Male	80	7555	7960	-1.081	0.2799
Female	118	12146	11741		
Combined	198	19701	19701		
Cognizance: Do you agree there is development and advancement in the field of dentistry?					
Male	80	7395.5	7960	-1.569	0.1166
Female	118	12305.5	11741		
Combined	198	19701	19701		
Cognizance: Do you agree a job or career from BDS will give you a steady employment?					
Male	80	7412	7960	-1.467	0.1425
Female	118	12289	11741		
Combined	198	19701	19701		

## Discussion

Dental interns frequently experience a blend of career aspirations, apprehensions and a need for guidance as they transition from their educational phase to professional practice. Dentistry was the first career choice for a balanced proportion of men and women. The motivation for studying dentistry primarily stemmed from personal interest, with no significant gender difference observed. Initial impressions upon joining BDS varied, with feeling satisfied being the most common among both genders. A significant majority of respondents expressed a desire to pursue further involvement in dentistry beyond undergraduate studies. There was a notable reliance on faculty for guidance, particularly among females, and a preference for specialization, especially among females.

In the present study, 22.2% of the participants chose dentistry as their first career choice, contrasting with the findings of the study conducted by Kannan and Kareem [15], where 66.3% chose dentistry as their primary career option.

Compared to the study findings of Rathod et al. [9] and Kabil et al. [16] where 5.8% and 67% of participants, respectively, chose dentistry based on personal interest, 31.8% of participants in the current study stated that their motivation for opting for this course was their personal interest in dentistry. Nonetheless, these results are in good agreement with the findings of the study by Sharma et al. [17] in which 44% of individuals chose dentistry out of personal interest.

In the present study, 47.9% of participants reported satisfaction upon starting their BDS course. This contrasts with Kannan et al.'s [15] findings, where 69.8% expressed satisfaction upon initially joining BDS.

In the present study, low pay scale (69.5%) and people neglecting oral health (56.1%) were the major limitations in dentistry as a profession according to women, whereas people neglecting oral health (43.8%) and increasing professional competition (43.7%) were the primary limitations. This was in contrast to the findings of the study conducted by Sajitha et al. [2], which showed job insecurity (27.9%) and low pay scale (30%) were the most prevalent concerns among men, while low pay (33.3%) was the most common concern among men.

In the present study, 45.4% of participants identified restricted job opportunities as the most significant challenge they might encounter after completing undergraduate studies. On the other hand, Rathod et al. [9] noted that 82% of participants attributed their challenges after BDS to a lack of funding, a sluggish pace of clinic expansion and a high level of competition.

In this study, more than half of the participants (108, 54.5%) expressed a desire to pursue postgraduate studies following the completion of their undergraduate course. This was similar to the study findings of Shetty et al. [18], which showed that 51% wanted to pursue postgraduation. This stands in contrast to the findings of the study conducted by Birajee et al. [1] and Xu et al. [19] where 91.2% and 95.8% of participants, respectively, expressed a desire for postgraduation after completing their undergraduate studies.

In the present study, postgraduation was favored by 65.7% of women and 34.2% of men. This differs from the findings of the study conducted by Saijtha et al. [2] where postgraduation was preferred by 61% of men and 37% of women.

According to Arhoma et al. [20] and Kapoor et al. [13], 67.3% and 81.4%, respectively, expressed a desire to opt for private practice, which contrasts sharply with the present study, where only 12.6% indicated a preference for private practice after completing their undergraduate course. The findings of the study conducted by Sharma et al. [17] showed that 26.9% wanted to opt for private practice. This significantly lower percentage may suggest a change in career preferences among recent dental graduates. Various factors could contribute to this shift, including concerns about financial uncertainty, the high cost of setting up a private clinic, lack of confidence in clinical skills and strong competition in the private sector. It may also reflect an increasing interest in pursuing structured postgraduate courses or salaried jobs that provide more security, professional guidance and better work-life balance. Furthermore, differences between institutions or regions - such as availability of clinical infrastructure, training in practice management and local job opportunities - could also have influenced these choices.

In the current study, 32.3% of participants sought career guidance from faculty, with 22.7% relying on seniors and friends. However, according to Sajitha et al. [2], 42.5% of individuals from the public sector relied on seniors, while 30.3% from the private sector relied on faculty for career guidance.

The comprehensive characterization of the study population provides valuable insights for understanding the background and socioeconomic context of dental interns, which can inform future research and interventions aimed at addressing relevant disparities and promoting equity in dental education and practice. Overall, the findings underscore the multifaceted interplay between individual motivations, societal perceptions and familial influences in shaping the vocational trajectories of dental interns, thereby emphasizing the importance of tailored career guidance and support programs in dental education institutions.

Dental interns harbor career aspirations such as specialization, practice ownership, or academic pursuits, yet face apprehensions about finances, clinical competence, and job market challenges. To support them, effective career guidance through mentorship, continuing education, financial planning and networking opportunities is essential.

The main strength of the study is that it explores gender differences in various aspects, such as career choice, motivation and initial impressions, providing insights into potential gender-related factors influencing dental education and practice. The identification of these factors provides a clearer understanding of the career development landscape of dental interns and highlights specific areas where support systems may be strengthened to better align with their professional needs.

One of the key limitations of this study is that, although the questionnaire incorporated both open- and close-ended items, it lacked comprehensive qualitative approaches such as interviews or focus group discussions. This limited the ability to gain deeper insights into participants' personal experiences and motivations. Additionally, the cross-sectional nature of the study captures data at a single point in time, preventing any conclusions about causal relationships. Future research could benefit from longitudinal designs that track students over an extended period, as well as mixed-method approaches that integrate quantitative data with detailed qualitative insights. Incorporating in-depth interviews or group discussions would help uncover the emotional and social dimensions influencing career decisions. Further investigation into the impact of academic mentorship and structured career guidance programs could also enhance understanding of how these factors shape vocational trajectories in dental education.

The study indicates a significant association between the occupation of the head of the family and vocational aspirations in dentistry. However, this association might be influenced by various socioeconomic factors such as access to education, familial expectations and social networks, which could confound the results. Therefore, it's important to acknowledge that these findings might not be generalizable to individuals from different socioeconomic backgrounds or regions with distinct social structures. As a quantitative cross-sectional study, it's important to acknowledge that various other variables may influence the perspectives of interns.

## Conclusions

This study provides valuable insights into the socioeconomic background, vocational aspirations, apprehensions and career guidance of dental interns, shedding light on potential areas for improvement in dental education and workforce development. The study indicated that both male and female dental interns showed a desire to further engage in dentistry beyond undergraduate studies, demonstrating a strong interest in continuing their education or career in the field.

The cognizance and apprehensions of dental interns reflect their evolving understanding of the dental profession and the challenges they may encounter as they transition from student to practicing professional. Addressing these concerns through mentorship, professional development opportunities, and supportive work environments can help interns navigate this pivotal phase of their dental careers with confidence and competence. By investing in career development support for dental interns, public health authorities can contribute to a stronger, more resilient dental workforce and better oral health outcomes for communities. Public health authorities, dental schools and professional organizations can collaborate to implement targeted interventions that address the identified challenges and capitalize on the shared aspirations of dental interns.

## Figures and Tables

**Figure 1:**
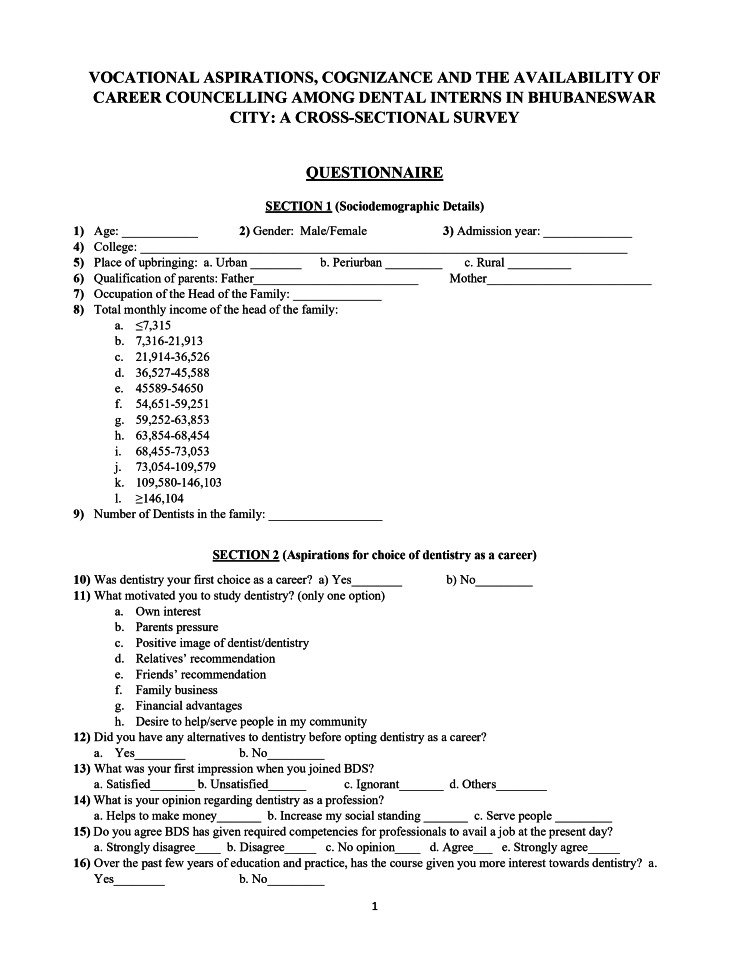
Self-structure questionnaire (1)

**Figure 2:**
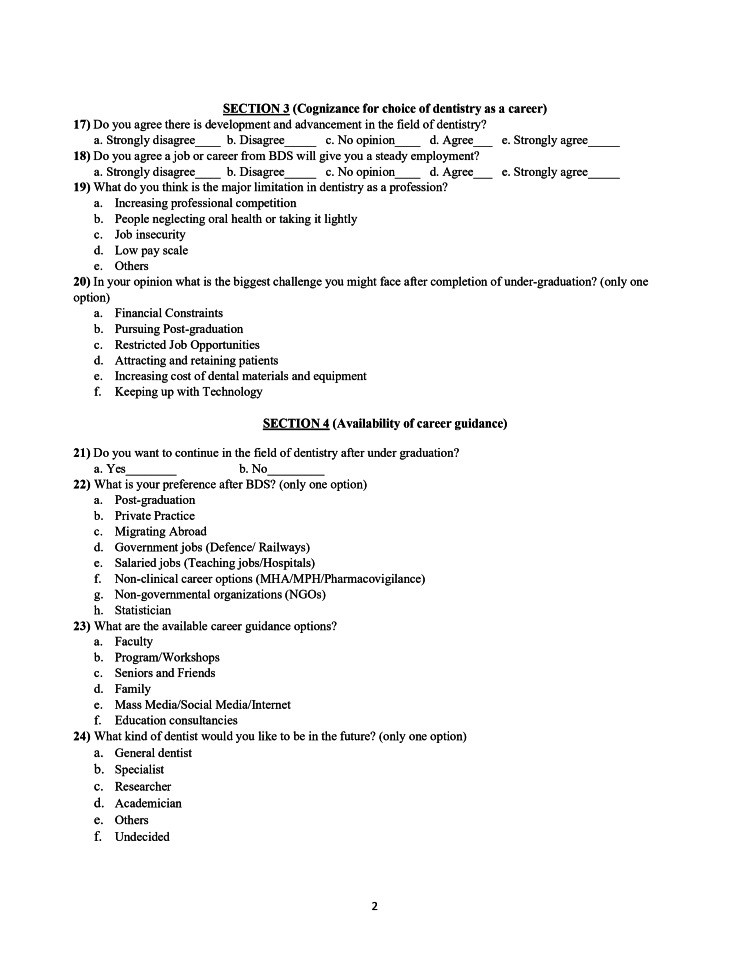
Self-structured questionnaire (2)
